# Erythrocytes Are an Independent Protective Factor for Vascular Cognitive Impairment in Patients With Severe White Matter Hyperintensities

**DOI:** 10.3389/fnagi.2022.789602

**Published:** 2022-02-18

**Authors:** Xi Tao, Hang Zhou, Danheng Mo, Wenjie Zhang, Zihan Chang, Yiheng Zeng, Yuqi Luo, Siyuan Wu, Wenjing Tang, Chen Yang, Qing Wang

**Affiliations:** ^1^Department of Neurology, Zhujiang Hospital, Southern Medical University, Guangzhou, China; ^2^Department of Neurological Rehabilitation, Hunan Provincial People’s Hospital, The First Affiliated Hospital of Hunan Normal University, Changsha, China

**Keywords:** cerebrovascular disease, erythrocyte, high-sensitivity C-reactive protein, prealbumin, retinol binding protein, vascular cognitive impairment, white matter hyperintensities

## Abstract

**Background and Purpose**: Hemoglobin is one of the main proteins in erythrocytes. There are significant correlations between low hemoglobin and white matter hyperintensities (WMH) and cognitive impairment. This study explored whether erythrocytopenia has predictive value for vascular cognitive impairment (VCI) in patients with WMH.

**Method**: We conducted a cross-sectional study of 302 patients, including 62 with cerebral small vessel disease and 240 with stroke. Basic demographic data and fasting blood were collected. First, all patients were divided into normal cognition (NC), mild VCI (mVCI), and severe VCI (sVCI) groups (subgroups later) based on cognitive behavior scores. Second, all patients were divided into mild WMH (mWMH) and severe WMH (sWMH) groups based on Fazekas scores. The differences in blood markers between different groups or subgroups with different cognitive levels were analyzed by univariate analysis. Then, binary logistic regression was used to analyze the diagnostic value of erythrocyte counts for VCI in the sWMH group, and ordinal logistic regression was used to analyze the predictive value of multiple variables for different cognitive levels.

**Results**: Univariate analysis showed that erythrocytes, hemoglobin, high-sensitivity C-reactive protein, retinol binding protein and prealbumin were potential blood markers for different cognitive levels in sWMH patients. Among them, erythrocytopenia has good predictive value for the diagnosis of mVCI (AUC = 0.685, *P* = 0.008) or sVCI (AUC = 0.699, *P* = 0.003) in patients with sWMH. Multivariate joint analysis showed that erythrocytes were an independent protective factor reducing the occurrence of VCI in patients with sWMH (OR = 0.633, *P* = 0.045). Even after adjusting for age, there was still a significant difference (*P* = 0.047).

**Conclusion**: Erythrocytes are an independent protective factor for VCI in patients with sWMH. Promoting hematopoietic function may have potential value for prevention of cognitive decline in patients with cerebrovascular disease.

## Introduction

Vascular cognitive impairment (VCI) is not only a common concomitant symptom in stroke patients but also an initial symptom in patients with cerebral small vessel disease (CSVD). Long-term and progressive cognitive impairment will develop into vascular dementia, which has become the type of dementia second only to Alzheimer’s disease (AD) in worldwide prevalence (Wang T. et al., 2020; Deng et al., 2021; Zhu et al., 2021a). The pathophysiological mechanisms underlying VCI are complex. The sudden physical mechanism of neural circuit rupture caused by macrovascular lesions (such as ischemic or hemorrhagic stroke; Xing and Bai, 2020; Yan et al., 2021) and the biochemical mechanisms (such as progressive oxidative stress, inflammatory responses, and abnormal immune regulation) triggered by small vascular lesions (Lin et al., 2021), are contributors. However, stroke events rarely occur in elderly patients independent of CSVD (Shao et al., 2020).

White matter hyperintensities (WMH), also known as white matter degeneration, are not only a typical imaging feature of CSVD but also an important biomarker of VCI (Cao S. et al., 2021). Our previous study found that fibrinogen was an independent risk factor for WMH in patients with CADASIL, but this correlation was not obvious in patients with sporadic CSVD (Guo et al., 2021). To date, studies examining blood biomarkers of VCI have mainly focused on vascular endothelial dysfunction (Cao Y. et al., 2021) and have been limited to a certain type of disease, such as CSVD or stroke (Zhu et al., 2019; Wang Y. et al., 2021). The detection of relevant blood biomarkers may be more persuasive and have greater predictive value when the heterogeneity of stroke lesion distribution in neural circuits is accounted for.

Erythrocytes contain a large number of important biologically active media (such as cholinesterase activity, amyloid-β, α-synuclein, hydroxyoctadecadienoic acid, oxidatively modified peroxiredoxin, magnesium, catalase activity, and superoxide dismutase), which play important roles in the central nervous system through circulatory functions of their carrier (von Bernhardi et al., 2005; Yoshida et al., 2009; Lauriola et al., 2018; Graham et al., 2019; Sitzia et al., 2020). With the chronic effects of vascular risk factors or stroke events, the imbalance in these components in erythrocytes or alterations in membrane surface receptors are not only closely related to cognitive impairment but also are important potential predictors of the occurrence and development of VCI (von Bernhardi et al., 2005; Yoshida et al., 2009; Lauriola et al., 2018; Graham et al., 2019; Sitzia et al., 2020). Studies have found that in elderly patients, severe chronic anemia can increase white matter stroke by 1.8 times, which is an independent risk factor for stroke exacerbation and cognitive impairment (Inzitari et al., 2008; Hao et al., 2013). Additionally, for young sickle and non-sickle cell anemia patients, reduced hemoglobin (Hb) can predict lower white matter volume and cognitive performance (Choi et al., 2019). Recently, studies have also found that the increase in the distribution width of erythrocytes is significantly related to severe WMH (Lee et al., 2016; Wang M. et al., 2020). There is a strong positive correlation between individual erythrocyte counts and Hb concentrations. Therefore, we speculate that there may be an association between erythrocyte levels and cognitive function in particular groups. The purpose of this study was to analyze whether the erythrocyte counts are associated with VCI based on the severity of WMH to provide strategies for the prevention of cognitive decline in patients with cerebrovascular diseases (CVDs).

## Materials and Methods

### Study Design

This was a prospective cross-sectional study at two centers (Hunan Provincial People’s Hospital and Zhujiang Hospital). This study was approved by the ethics committee of Hunan Provincial People’s Hospital of Hunan Normal University (Human Ethics Number: 2021SRERN60) and conducted in accordance with the principles outlined in the revised Declaration of Helsinki of 1975 and the National Institutes of Health Human Subjects Policies and Guidelines released in 1999. All participants signed the informed consent form for blood sample inspection and questionnaire survey. For patients with severe cognitive impairment, a family member’s representative was required.

### Participant Groups

**Figure 1:**
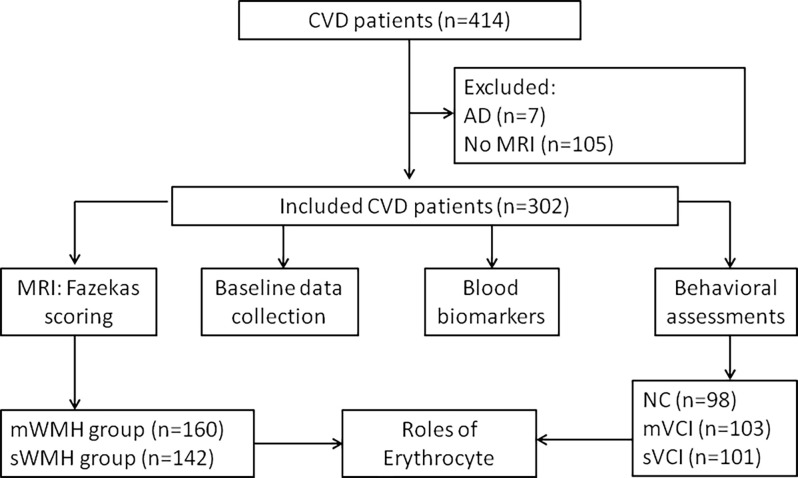
Study flow chart. Four-hundred and fourteen patients with CVD were enrolled, seven patients with suspected AD and 105 patients without MRI data were excluded, and 302 patients were included. Baseline demographic data, blood biomarkers, MRI images, and behavioral scores were collected. The patients were divided into mWMH and sWMH groups based on Fazekas scoring. A model was constructed to predict the diagnostic value of erythrocytes for VCI. AD, Alzheimer’s disease; CVD, cerebrovascular disease; mVCI, mild vascular cognitive impairment; mWMH, mild white matter hyperintensities; NC, normal cognition; sWMH, severe white matter hyperintensities; sVCI, severe vascular cognitive impairment; VCI, vascular cognitive impairment.

From May 2020 to July 2021, we collected basic information on 414 patients with CVD. Among them, seven patients with possible AD and 105 patients with head CT results only were excluded. Finally, 302 patients with brain MRI results were enrolled, including 62 CSVD and 240 stroke patients (Figure 1).

All patients with CSVD were diagnosed after admission for some reason, such as dizziness, headache, poor blood sugar control, hypertension, memory impairment, anxiety, or depressive psychological disorders. Then, typical lacunar infarction, white matter degeneration, cerebral microhemorrhage, or cerebral atrophy was revealed by 1.5T brain MRI examination. Stroke patients had clear symptoms of neurological impairment, such as slurred speech, swallowing disorders, limb weakness, or sensory disturbances. For patients with ischemic stroke, the symptoms needed to last for more than 72 h. Examination of routine MRI sequences was needed. However, for hemorrhagic stroke, an MRI examination was required after the absorption of the hemorrhage.

The inclusion criteria were as follows: (1) diagnosis with CSVD or unilateral cerebral stroke; (2) consciousness; (3) stable vital signs; and (4) cooperation during behavioral examinations. The exclusion criteria were as follows: (1) nonvascular cognitive impairment before stroke; (2) severe aphasia; (3) infection within 2 weeks before evaluation; (4) severe liver (alanine aminotransferase >200 U/L) or kidney [glomerular filtration rate <30 ml/(min × 1.73 m^2^)] insufficiency; and (5) refusal to participate in this study.

### Clinical Characterization

The basic demographic information of all patients with cognitive impairments was collected, including age, sex, body mass index (BMI), educational background, history of stroke, coronary artery disease, atrial fibrillation, diabetes mellitus, hypertension, and history of smoking and drinking. A history of stroke was based on hospitalization due to stroke events with or without sequelae. Coronary heart disease (CHD), diabetes mellitus, and hypertension were diagnosed before or after admission.

### Behavioral Assessments

Based on the 2018 guidelines from the Vascular Impairment of Cognition Classification Consensus Study (VICCCS; Skrobot et al., 2018) and the 2019 diagnosis and treatment framework of vascular cognitive impairment in China, we used the Mini-Mental State Examination (MMSE) scale to evaluate the overall cognitive function of all patients (Zhu et al., 2019). The following scoring thresholds for cognitive impairment (dementia) were adopted based on education background: illiteracy, ≤17 points; 1–6 years, ≤19 points; and ≥7 years, ≤24 points. To assess cognitive subdomains, we used the Boston Naming Test (BNT) to evaluate language function (Nizamutdinov et al., 2021), the Hopkins Verbal Language Learning Lest-Revised (HVLT-R) to evaluate learning and memory abilities (Lan et al., 2021), the Clock Drawing Test (CDT) to evaluate visuospatial abilities (Schejter-Margalit et al., 2021), and the Trail-Making Test (TMT-A) to evaluate executive function (Nizamutdinov et al., 2021). In addition, activities of daily living were evaluated by the modified Barthel index (MBI; Qu et al., 2021). Severe VCI (sVCI) was defined as the threshold of dementia with MMSE scores, and mild VCI (mVCI) was defined as an abnormality in one of the tests, i.e., the BNT, HVLT, CDT, or TMT-A, even if the MMSE score was normal. Normal cognition (NC) was defined as having scores in MMSE and all four subdomains that were normal (Skrobot et al., 2018).

### Blood Biomarker Examination

Blood samples of all patients were collected at 6–7 a.m the day after fasting for at least 8 h. Two milliliters of EDTA-anticoagulated whole blood were used for routine blood tests (automated hematology analyzer, XN-10, JAPAN) that included erythrocyte, lymphocyte, and platelet counts and a hemoglobin concentration determination. Five milliliters of blood containing coagulant was used for common biochemical examination (automatic analyzer, HITACHI 7600, JAPAN) that included retinol binding protein (RBP), creatinine (Cr), urea nitrogen (UN), β_2_ microglobulin (β_2_-M), alkaline phosphatase (ALP), triglycerides (TG), high-density lipoprotein cholesterol (HDL-C), low-density lipoprotein cholesterol (LDL-C), very low-density lipoprotein cholesterol (VLDL-C), homocysteine (Hcy), high-sensitivity C-reactive protein (hs-CRP), ischemia-modified albumin (IMA) and prealbumin (PA). All the indicators were tested using commercial kits, which were operated by qualified professionals in accordance with the specifications.

### MRI Scan and Analysis

**Figure 2:**
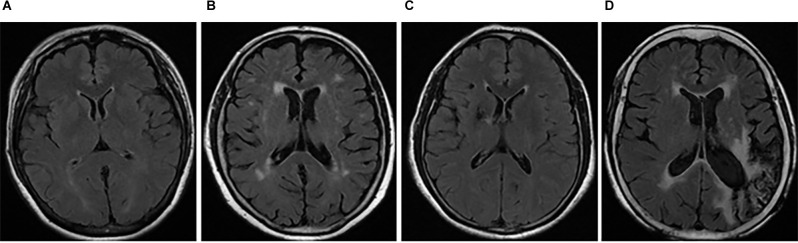
Typical WMH images in patients with CSVD and stroke. T2WI-FLAIR MRI sequences were analyzed in the patients with CSVD and/or stroke. The total Fazekas scores of typical patients with CSVD were 1 **(A)** and 4 **(B)**, and those of typical stroke patients with infarction of the right basal ganglia and left occipital lobe were 1 **(C)** and 4 **(D)**, respectively. Panels **(A,C)** were identified as mWMH, and **(B,D)** as sWMH. CSVD, cerebral small vessel disease; mWMH, mild white matter hyperintensities; NC, normal cognition; sWMH, severe white matter hyperintensities.

Images including T1WI, T2WI, and T2WI-FLAIR sequences were acquired using a 1.5T scanner (Siemens magnet on trio, A Tim System, Germany). WMH was defined as a hyperintensity in the white matter regions observed on the T2WI-FLAIR sequence, including periventricular WMH (PWMH) and deep WMH (DWMH; Fazekas et al., 1987; Cao S. et al., 2021). DWI sequence detection was needed for patients with acute CVD (<2 weeks) to rule out the effects of focal edema and space-occupation on WMH. Based on standard Fazekas scoring (0, normal; 1, mild; 2, moderate; 3, severe), the severity of PWMH and DWMH were separately scored (Fazekas et al., 1987). For stroke patients, WMH in the contralateral hemisphere of the lesion was the focus of identification. Then, the sum of Fazekas scores for PWMH and DWMH was calculated to obtain a total score. Those with a total score <3 points were categorized as mild WMH (mWMH), and those with a total score ≥3 points were categorized as severe WMH (sWMH; Figure 2).

In addition, lesions larger than 1.5 cm in diameter were manually counted, including different brain lobes, the basal ganglia, and the brainstem. All imaging evaluations were performed by two experienced senior neurologists. Controversial issues were resolved through consultation.

### Statistical Analysis

All data were analyzed using SPSS 24.0 statistical software. (1) For continuous variables such as blood biomarkers, age, and BMI, exploratory analyses were initially conducted. All the compared data with a normal distribution are presented as the mean ± standard deviation (SD), otherwise, the data are presented as the median (interquartile range). (2) For continuous variables in the mWMH and sWMH groups, two independent sample *t*-tests were used for normally distributed data, and Mann–Whitney *U* nonparametric tests were used for nonnormally distributed data. (3) For the NC, mVCI, and sVCI groups (subgroups), one-way ANOVA was performed among the three groups if the data were normally distributed. For *post hoc* test, Tukey tests were used when equal variances were assumed, and otherwise, Dunnett’s T3 tests were used when equal variances were not assumed. Furthermore, if one of the variables among the three groups was nonnormally distributed, the Kruskal–Wallis *H* test was used followed by Bonferron adjusted. Then, two independent sample *t*-tests or Mann–Whitney *U*-tests were used to compare two groups based on the data distribution characteristics. (4) Chi square tests were used to compare percentages between groups. (5) In the sWMH group, we took the patients with NC as the control and analyzed the diagnostic value of univariate erythrocytes for mVCI and sVCI by binary logistic regression. (6) The presence and absence of parietal lobe lesions and atrial fibrillation were used as factors, and the continuous variables erythrocyte, hs-CRP, RBP, and PA levels were used as covariates. Ordinal logistic regression was used to analyze the predictive value of these variables at different cognitive levels and was adjusted for the confounding factors of age and (or) sex.

## Results

### Demographic Characteristics

Three hundred and two patients were included in this study, including 240 patients with stroke and 62 patients with CSVD. Twelve people had a history of stroke. Based on the classification standard for VCI, they were divided into the NC group (*n* = 98), mVCI group (*n* = 103) and sVCI group (*n* = 101). The average ages of those in the VCI groups were significantly higher than the age of those in the NC group (all *P* < 0.001), while the BMI of those in the sVCI group was significantly lower than those in the other groups (*P* = 0.005). There was a significant difference in the educational background among the three groups (*P* = 0.016). Interestingly, the proportion of CSVD in the sVCI group was significantly lower (*P* = 0.001), while the proportion of atrial fibrillation was clearly higher (*P* = 0.004). Also, we found that there was a significant difference between CSVD and stroke groups with different cognitive impairments (*P* = 0.001). The proportion of sWMH was higher in the stroke group (122/240) than that in the CSVD group (20/62), *P* = 0.009 (data not shown in table). There were no significant differences among the three groups in sex, ischemic or hemorrhagic stroke, history of stroke, duration of stroke, hypertension, diabetes mellitus, coronary artery disease, or history of smoking and alcohol intake (Supplementary Table 1).

### Comparison of Blood Markers and WMH Among Different Groups

Compared with the NC group, erythrocytes decreased in mVCI (*P* = 0.027) and sVCI (*P* = 0.000) groups, while Hb decreased in sVCI (*P* = 0.002) group, serum β_2_-M increased significantly in mVCI (*P* = 0.005), and sVCI (*P* = 0.011) groups. However, there were no differences in the comparisons of the three markers between the mVCI and sVCI groups. Compared with the NC or mVCI group, PA (*P*_NC_ = 0.001 and *P*_mVCI_ = 0.025) significantly decreased in sVCI group. With the aggravation of cognitive impairment, although lymphocytes and RBP decreased and IMA increased among the three groups, the differences were limited to the comparison between lymphocytes (*P* = 0.001) or RBP (*P* = 0.044) or IMA (*P* = 0.013) in sVCI group and NC group. Unlike all other markers, hs-CRP levels gradually increased with the aggravation of cognitive impairment, and there were significant differences among the three (*P* < 0.001) and between any two (all *P* < 0.05) groups. There were no significant differences in the comparisons of Hcy, Cr, UN, ALP, TG, HDL-C, LDL-C and, VLDL-C levels among the groups (Supplementary Table 2).

Based on standard Fazekas scoring, we identified paraventricular and subcortical WMH, evaluated the scores, and accumulated the total scores from every T2WI-FLAIR image. The total WMH scores, PWMH and DWMH scores gradually increased across the three groups (all *P* < 0.001). However, pairwise comparisons of Fazekas scores of different cognitive levels were limited to NC and mVCI (all *P* < 0.05) or sVCI (all *P* < 0.001) group, while the differences between mVCI and sVCI were not statistically significant (Supplementary Table 2).

### Comparison of Behavioral Scores Among Different Groups

All patients completed the MMSE, MBI, CDT, BNT, and HVLT-R tests. From the NC through the sVCI groups, these scores gradually decreased. The differences among the three groups were very significant (all *P* < 0.001). The patients with abnormal TMT-A results or who were unable to complete the test were recognized as having an abnormal executive function. Due to data issues, the results from the TMT-A were not included in the final statistical analysis (Supplementary Table 2).

### Demographic Characteristics of the mWMH and sWMH Groups

Based on the total WMH scores, 302 patients were divided into mWMH (*n* = 160) and sWMH (*n* = 142) groups. In the mWMH group, the patients with mVCI (*P* < 0.001) or sVCI (*P* = 0.003) were older than those with NC, while there were no significant differences in sex, educational background, BMI, classification of CVD, or vascular risk factors among the three subgroups. In the sWMH group, the proportion of atrial fibrillation in the sVCI subgroup was significantly higher than that in the NC and mVCI subgroups (*P* = 0.026). Interestingly, the proportion of smoking history in the sVCI subgroup was the lowest (*P* = 0.004). There were no significant differences in other variables among the three subgroups (Table 1).

**Table 1:** Basic demographic data of the mWMH and sWMH groups with different cognitive levels.

Variable	Groups	NC	mVCI	sVCI	*F/Χ^2^/H*	*P*
Age, years^a^	mWMH	55.74 ± 11.89	64.51 ± 12.19	63.00 ± 11.73	9.351	0.000***
	sWMH	24.16 ± 2.73	24.19 ± 2.66	23.19 ± 3.46	1.649	0.196
BMI (kg/m^2^)^a^	mWMH	65.27 ± 11.05	67.88 ± 11.01	69.66 ± 10.16	1.608	0.204
	sWMH	24.33 ± 3.11	23.00 ± 3.18	22.55 ± 3.37	2.764	0.067
Sex (male)^b^	mWMH	50 (69.44)	33 (64.71)	21 (56.76)	1.732	0.421
	sWMH	23 (88.46)	37 (71.15)	41 (64.06)	5.360	0.069
Education^b^						
0		0 (0.00)	0 (0.00)	1 (2.70)		
<6	mWMH	8 (11.11)	14 (27.45)	7 (18.92)	8.355	0.079
≥7		64 (88.89)	37 (72.55)	29 (78.38)		
0		0 (0.00)	5 (9.62)	5 (7.81)		
<6	sWMH	5 (19.23)	10 (19.23)	14 (21.88)	4.564	0.335
≥7		21 (80.77)	37 (71.15)	45 (70.31)		
Stroke						
Ischemic	mWMH	44 (61.11)	29 (56.86)	26 (70.27)	1.666	0.435
stroke^b^	sWMH	16 (61.54)	35 (67.31)	50 (78.13)	3.060	0.217
Hemorrhagic	mWMH	7 (9.72)	7 (13.73)	8 (21.62)	2.918	0.232
stroke^b^	sWMH	4 (15.38)	9 (17.31)	12 (18.75)	0.152	0.927
Disease	mWMH	1.45 (3.80)	1.50 (5.70)	1.00 (2.15)	1.682	0.431
duration^c^	sWMH	3.20 (35.50)	1.35 (3.34)	3.20 (11.53)	1.979	0.372
CSVD^b^	mWMH	22 (30.56)	16 (31.37)	4 (10.81)	5.936	0.051
	sWMH	7 (26.92)	8 (15.38)	5 (7.81)	5.412	0.067
History of stroke^b^	mWMH	1 (1.39)	2 (3.92)	1 (2.70)	0.801	0.670
	sWMH	2 (7.69)	2 (3.85)	4 (6.25)	0.581	0.748
Hypertension^b^	mWMH	53 (73.61)	40 (78.43)	32 (86.49)	2.375	0.305
	sWMH	21 (80.77)	41 (78.85)	52 (81.25)	0.110	0.947
Diabetes mellitus^b^	mWMH	29 (40.28)	17 (33.33)	14 (37.84)	0.617	0.735
	sWMH	8 (30.77)	22 (42.31)	27 (42.19)	1.164	0.559
CHD^b^	mWMH	10 (13.89)	14 (27.45)	10 (27.03)	4.241	0.120
	sWMH	10 (38.46)	13 (25.00)	22 (34.38)	1, 839	0.399
Atrial fibrillation^b^	mWMH	2 (2.78)	1 (1.96)	2 (5.41)	0.817	0.665
	sWMH	1 (3.85)	2 (3.85)	11 (17.19)	7.277	0.026*
Smoking^b^	mWMH	23 (31.94)	16 (31.37)	12 (32.43)	0.011	0.994
	sWMH	16 (61.54)	17 (65.38)	16 (25.00)	11.042	0.004**
Alcohol intake^b^	mWMH	16 (22.22)	7 (13.73)	6 (16.22)	1.571	0.456
	sWMH	7 (26.92)	7 (13.46)	12 (18.75)	2.054	0.358

*P < 0.05,**P < 0.01,***P < 0.001. ^a^Shown as Mean ± standard deviation. ^b^Shown as n (%). ^c^Shown as median (IQR). *Abbreviations*: BMI, body mass index; CHD, Coronary heart disease; CSVD, cerebral small vessel disease; mVCI, mild vascular cognitive impairment; mWMH, mild white matter hyperintensities; NC, normal cognition; SD, standard deviation; sVCI, severe vascular cognitive impairment; sWMH, severe white matter hyperintensities.

### Comparison of the Proportion of Lesions Among the Three Cognitive Levels Based on WMH Classification

We identified and counted the presence of brain atrophy and all lesions larger than 1.5 cm in diameter in MRI images. Overall, the proportion of brain atrophy (*P* < 0.001) and lesion involvement [such as frontal lobe (*P* = 0.011), parietal lobe (*P* < 0.001), temporal lobe (*P* = 0.002), occipital lobe (*P* = 0.008), basal ganglia (*P* = 0.014), insular lobe (*P* = 0.044) and corpus callosum (*P* = 0.044) among the three groups gradually increased as cognitive impairment was aggravated. In the mWMH group, the thalamus (*P* = 0.037) and hippocampus (*P* = 0.023) replaced the basal ganglia and corpus callosum, and the proportions of other affected lesions and brain atrophy, as the whole, were also significantly different (all *P* < 0.05). However, it is interesting to note that in the sWMH group, the proportion of parietal lobe lesions (*P* = 0.002) showed a progressive increase with cognitive severity, while the number of lesions in the corona radiata (*P* = 0.010) was the highest in the mVCI subgroup (26.92%; Supplementary Table 3).

### Multivariable Comparison Between mWMH and sWMH Groups

Compared with the mWMH group, the sWMH group was significantly older (*P* < 0.001), had obviously higher serum hs-CRP (*P* = 0.024), Hcy (*P* = 0.016) and β_2_-M (*P* < 0.001) levels, and significantly lower BMI (*P* = 0.013), Hb (*P* = 0.033) and PA (*P* = 0.003). However, there were no significant differences in erythrocyte and lymphocyte counts, RBP, Cr, HDL-C, LDL-C, or IMA between the two groups (Table 2).

In addition, not only MMSE scores (*P* < 0.001) and every cognitive subfield score (*P* < 0.001) but also MBI scores (*P* < 0.001) were significantly lower in the sWMH group than in the mWMH group (Table 2).

**Table 2:** Comparison of variables between the mWMH and sWMH groups.

Variables	mWMH (*n* = 160)	sWMH (*n* = 142)	*F/Z*	*P*
Age (years)^b^	62.00 (17.00)	68.00 (14.50)	5.433	0.000***
BMI (kg/m^2^)^a^	23.94 ± 2.90	23.04 ± 3.30	3.334	0.013*
Erythrocyte (× 10^12^/L)^b^	4.23 (0.76)	4.21 (0.73)	1.047	0.295
Hb (g/L)^a^	132.45 ± 16.01	128.20 ± 18.34	2.340	0.033*
Platelets (× 10^9^/L)^b^	215.00 (82.25)	219.00 (75.25)	0.292	0.770
Lymphocytes(× 10^9^/L)^b^	1.69 (0.80)	1.57 (0.74)	1.186	0.235
hs-CRP (mg/L)^b^	1.83 (3.72)	2.75 (7.14)	2.263	0.024*
Hcy (μmol/L)^b^	13.85 (6.24)	15.22 (6.03)	2.409	0.016*
RBP (mg/L)^b^	37.80 (13.43)	36.20 (13.95)	1.502	0.133
Cr (μmol/L)^b^	65.00 (23.75)	69.00 (27.00)	1.247	0.212
UN (mmol/L)^ b^	5.31 (2.13)	5.20 (1.92)	0.892	0.372
β_2_-M (mg/L)^b^	2.10 (0.74)	2.40 (0.93)	3.655	0.000***
ALP (U/L)^b^	68.50 (26.00)	71.50 (21.75)	0.982	0.326
TG (mmol/L)^b^	1.38 (0.89)	1.27 (0.84)	1.710	0.087
HDL-C (mmol/L)^b^	1.04 (0.35)	0.99 (0.41)	0.953	0.340
LDL-C (mmol/L)^b^	2.35 (1.41)	2.28 (1.14)	1.250	0.211
VLDL-C (mmol/L)^b^	0.40 (0.26)	0.35 (0.22)	1.430	0.153
IMA (U/mL)^a^	77.16 ± 4.14	77.78 ± 4.13	0.035	0.195
PA (mg/L)^a^	266.33 ± 62.06	244.54 ± 66.41	2.363	0.003**
MMSE^b^	27.00 (5.00)	24.00 (10.00)	5.151	0.000***
CDT^b^	4.00 (1.00)	3.00 (2.00)	4.681	0.000***
BNT^b^	22.00 (4.00)	19.00 (9.25)	6.111	0.000***
HVLT-R^b^	18.00 (6.00)	14.00 (8.00)	5.478	0.000***
MBI^b^	90.00 (45.00)	67.50 (46.25)	3.950	0.000***

*P < 0.05,**P < 0.01,***P < 0.001. ^a^Shown as Mean ± standard deviation. ^b^Shown as median (IQR). *Abbreviations*: ALP, alkaline phosphatase; BNT, Boston Naming Test; β_2_-M, β_2_ microglobulin; CDT, Clock Drawing Test; Cr, creatinine; Hb, hemoglobin; Hcy, homocysteine; HDL-C, high-density lipoprotein cholesterol; hs-CRP, high-sensitivity C-reactive protein; HVLT-R, Hopkins Verbal Language Learning Lest-Revised; IMA, ischemia modified albumin; LDL-C, low-density lipoprotein cholesterol; MBI, modified Barthel index; MMSE, Mini Mental State Examination; mWMH, mild white matter hyperintensities; PA, prealbumin; RBP, retinol binding protein; sWMH, severe white matter hyperintensities; TG, triglycerides; TMT-A, Trail Making Test; UN, urea nitrogen; VLDL-C, very low-density lipoprotein cholesterol.

### Multivariate Comparison of Blood Markers Among Three Different Cognitive Levels in the mWMH and sWMH Groups

**Figure 3:**
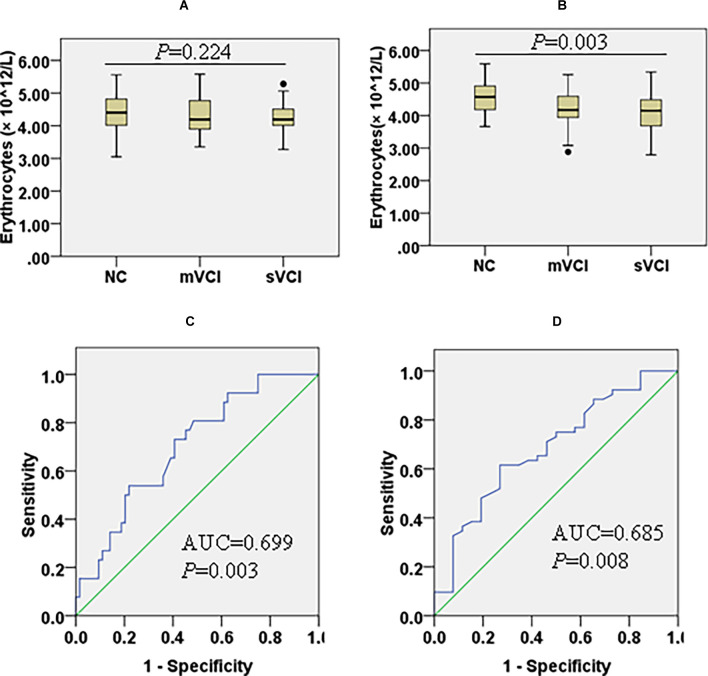
Box diagram of erythrocytes in the mWMH and sWMH groups and ROC curves to predict the diagnostic value of erythrocytes for mVCI and sVCI in the sWMH group. Comparison of erythrocyte counts among patients with three cognitive levels in the mWMH **(A)** (*H* = 2.993, *P* = 0.224) and sWMH **(B)** (*F* = 6.094, *P* = 0.003) groups. **(C)** Diagnostic value of erythrocytes to sVCI in the sWMH group [AUC = 0.699, *P* = 0.003, 95% CI (0.586, 0.812)]. **(D)** Diagnostic value of erythrocytes to mVCI in the sWMH group [AUC = 0.685, *P* = 0.008, 95% CI (0.561, 0.810)]. AUC, area under the ROC curve; mVCI, mild vascular cognitive impairment; mWMH, mild white matter hyperintensities; ROC, receiver operating characteristic; sWMH, severe white matter hyperintensities; sVCI, severe vascular cognitive impairment.

In the mWMH group, compared with the NC subgroup, the lymphocyte count (*P* = 0.003) decreased and the level of hs-CRP (*P* = 0.003) increased in the sVCI subgroup. Compared with the mVCI subgroup, lymphocyte count (*P* = 0.020), serum Cr (*P* = 0.031), and HDL-C (*P* = 0.014) were significantly decreased in the sVCI subgroup. There were no significant differences in the comparison of the remaining markers (Figure 3A, Supplementary Table 4).

In the sWMH group, compared with the NC subgroup, erythrocyte counts (*P*_mVCI_ = 0.026, *P*_sVCI_ = 0.002) were significantly lower in the mVCI and sVCI subgroups (Figure 3B), but Hb was significantly lower only in the sVCI subgroup (*P* = 0.010). Compared with the mVCI subgroup, PA (*P* = 0.027) in the sVCI subgroup significantly decreased, while hs-CRP increased with the aggravation of cognitive impairment, and the difference was mainly limited to the comparison between NC (*P* = 0.004) or mVCI subgroup (*P* = 0.024) and sVCI subgroup. There were no significant differences in the comparisons of the remaining variables (Table 3).

**Table 3:** Comparison of various biomarkers of different cognitive levels in the sWMH group.

Variable	NC (*n* = 26)	mVCI (*n* = 52)	sVCI (*n* = 64)	*F/H*	*P*	Tukey/Bonferron adjusted		
						NC *vs*. mVCI	NC *vs*. sVCI	mVCI *vs*. sVCI
Erythrocyte (× 10^12^/L)^a^	4.56 ± 0.51	4.21 ± 0.50	4.12 ± 0.60	6.094	0.003**	0.026*	0.002**	0.606
Hb (g/L)^a^	137.00 ± 16.70	128.19 ± 16.30	128.20 ± 18.34	4.401	0.014*	0.105	0.010*	0.539
Platelets (× 10^9^/L)^b^	208.00 (67.50)	220.00 (79.50)	223.00 (86.25)	2.742	0.254	/	/	/
Lymphocytes(×10^9^/L)^b^	1.97 (0.72)	1.54 (0.72)	1.55 (0.63)	5.718	0.057	/	/	/
hs-CRP (mg/L)^b^	1.22 (2.41)	2.20 (4.01)	4.68 (10.52)	12.803	0.002**	0.895	0.004**	0.024*
Hcy (μmol/L)^b^	15.68 (6.43)	14.49 (5.35)	15.64 (7.27)	1.840	0.398	/	/	/
RBP (mg/L)^b^	40.00 (9.58)	38.40 (16.30)	33.35 (14.20)	6.201	0.045*	1.000	0.117	0.123
Cr (μmol/L)^b^	76.10 (24.75)	67.00 (31.50)	68.00 (23.00)	2.853	0.240	/	/	/
UN (mmol/L)^ b^	5.17 (1.32)	5.12 (1.87)	5.37 (2.39)	1.195	0.550	/	/	/
β_2_-M (mg/L)^b^	2.12 (0.94)	2.50 (0.95)	2.40 (0.91)	3.401	0.183	/	/	/
ALP (U/L)^b^	65.00 (25.50)	70.00 (23.75)	73.00 (23.00)	2.002	0.368	/	/	/
TG (mmol/L)^b^	1.45 (1.13)	1.29 (0.75)	1.25 (0.82)	0.034	0.983	/	/	/
HDL-C (mmol/L)^b^	1.08 (0.36)	0.95 (0.39)	0.99 (0.43)	1.383	0.501	/	/	/
LDL-C (mmol/L)^b^	2.40 (1.40)	2.07 (1.09)	2.40 (1.15)	3.296	0.192	/	/	/
VLDL-C (mmol/L)^b^	0.37 (0.28)	0.33 (0.20)	0.36 (0.26)	0.902	0.637	/	/	/
IMA (U/mL)^a^	77.36 ± 4.01	77.18 ± 4.23	78.43 ± 4.06	1.479	0.232	/	/	/
PA (mg/L)^a^	259.44 ± 50.11	258.62 ± 67.28	227.05 ± 68.15	4.229	0.016*	0.998	0.085	0.027*

*P < 0.05,**P < 0.01. ^a^Shown as mean ± standard deviation. ^b^Shown as median (IQR). *Abbreviations*: ALP, alkaline phosphatase; β_2_-M, β_2_ microglobulin; Cr, creatinine; Hb, hemoglobin; Hcy, homocysteine; HDL-C, high-density lipoprotein cholesterol; hs-CRP, high-sensitivity C-reactive protein; IMA, ischemia modified albumin; LDL-C, low-density lipoprotein cholesterol; mVCI, mild vascular cognitive impairment; NC, normal cognition; PA, prealbumin; RBP, retinol binding protein; sWMH, severe white matter hyperintensities; sVCI, severe vascular cognitive impairment; TG, triglycerides; UN, urea nitrogen; VLDL-C, very low-density lipoprotein cholesterol.

### Diagnostic Value of Erythrocytes in the sWMH Group for VCI

In the sWMH subgroup, sVCI was used as the diagnostic target, and a ROC curve was constructed (Figure 3C). We found that erythrocytes had significant diagnostic value regarding cognitive levels after cerebrovascular injury [AUC = 0.699, *P* = 0.003, 95% CI (0.586, 0.812)], and the cutoff was 4.30 g/L, with a sensitivity of 73.08% and specificity of 59.38%. With mVCI as the diagnostic target, the diagnostic value was still clear [AUC = 0.685, *P* = 0.008, 95% CI (0.561, 0.810)], and the cutoff was 4.31 g/L, with a sensitivity of 73.08% and specificity of 61.54% (Figure 3D).

### Multivariate Prediction of Different Cognitive Levels With Ordinal Logistic Regression

Taking the differential variables in the sWMH group as a reference, the presence of parietal lobe lesions, atrial fibrillation, erythrocytes, hs-CRP, RBP, and PA were included in the model. The results showed that erythrocytes were an important protective factor for cognitive function in the sWMH group (OR = 0.633, *P* = 0.045). After adjusting for age, erythrocytes still had a significant protective value (*P* = 0.047), but after adjusting for sex, the independent protective value of erythrocytes was not significant (*P* = 0.067). Additionally, the prediction model for cognition in the mWMH group was constructed with the same six variables. The results showed that parietal lobe lesions were the main risk factor (OR = 1.209, *P* = 0.000; Table 4).

**Table 4:** Multivariate prediction of different cognitive levels with ordinal logistic regression.

Variables	mWMH		sWMH			
	OR (95% CI)	*P*	OR (95% CI)	*P*	^#^Adjusted OR (95% CI)	*P*
Parietal lobe	1.209 (0.381, 3.834)	0.000***	1.525 (0.773, 3.010)	0.224	1.522 (0.769, 3.010)	0.228
Atrial fibrillation	2.575 (1.533, 4.328)	0.748	2.762 (0.848, 8.998)	0.092	2.776 (0.847, 9.098)	0.092
Erythrocyte	0.685 (0.448, 1.045)	0.079	0.633 (0.405, 0.989)	0.045*	0.631 (0.401, 0.994)	0.047*
hs-CRP	1.022 (0.993, 1.051)	0.145	1.017 (0.994, 1.041)	0.148	1.017 (0.994, 1.041)	0.152
RBP	1.013 (0.980, 1.046)	0.447	1.012 (0.976, 1.049)	0.525	1.012 (0.975, 1.050)	0.526
PA	0.996 (0.990, 1.002)	0.227	0.997 (0.989, 1.004)	0.364	0.997 (0.989, 1.004)	0.390

*P < 0.05, ***P < 0.001. ^#^Indicates age adjusted. *Abbreviations*: hs-CRP, high-sensitivity C-reactive protein; mWMH, mild white matter hyperintensities; PA, prealbumin; RBP, retinol binding protein; sWMH, severe white matter hyperintensities.

## Discussion

This study provided the following new findings: (1) Blood-derived erythrocyte, Hb, lymphocyte, hs-CRP, RBP, β_2_-M, IMA, and PA levels and WMH may be potential markers to predict different cognitive levels in patients with CVD. (2) Hb, hs-CRP, Hcy, β_2_-M, and PA levels may have distinguishing values with regard to WMH with different severities. (3) Hs-CRP is a common potential marker of different cognitive levels in patients with mWMH or sWMH, while erythrocyte, Hb, RBP, and PA levels are unique to the latter. (4) Erythrocytes have good predictive value for the diagnosis of VCI in patients with sWMH and are an independent protective factor that reduced the occurrence of VCI in those patients.

Serum biomarkers play an important role in predicting the occurrence and development of VCI (Li et al., 2020; Wang J. H. et al., 2021). Compared with a single marker, the combined detection of multiple markers can provide incremental improvements in predicting VCI from different pathological pathways. Through univariate analyses, this study found that five proteins, RBP, β_2_-M, IMA, PA, and hs-CRP, and lymphocyte counts may be related to VCI. RBP is mainly derived from the liver and participates in vitamin transport. The decrease in its expression has been closely related to the cognitive impairment of patients with AD (Jung et al., 2008) and spinocerebellar ataxia type 2 (Swarup et al., 2013). β_2_-M is secreted by lymphocytes and platelets, and its increased expression is related to AD (Dominici et al., 2018) and poor cognition in hemodialysis patients (Miller et al., 2021). Unlike RBP and β_2_-M, which reflect the function of renal tubules, IMA is a modification of albumin that is produced after myocardial ischemia. Recent research found that, as one of the products of oxidative stress, elevated plasma IMA is related to cognitive deficits in the early stage of AD (Du et al., 2019; Gündüztepe et al., 2020). PA (also known as transthyretin) is also secreted by the liver and has been widely mentioned in predicting early cognitive deficits in AD (Tien et al., 2019) and as a target of cognitive interventions (Saponaro et al., 2020). However, these four proteins are far less widely mentioned in VCI research than hs-CRP (Slevin et al., 2020; Wanggong et al., 2021). This study found that RBP, β_2_-M, IMA, and PA may be potential plasma markers for the diagnosis of VCI, which has rarely been reported in previous studies. In addition, lymphocyte count and classification are signs of the body’s immune activity. With the discovery of the value of the neutrophil-lymphocyte ratio in the long-term predictive value of post-stroke cognitive impairment (Lee et al., 2021), the damage to the vascular nerve unit and cognitive dysfunction mediated by immune responses need to be further studied.

In addition, this study found that Hb and erythrocyte counts may be associated with VCI through univariate analyses. As mentioned earlier, the reduction in Hb, which reflects anemia, is an important risk factor for white matter stroke and cognitive impairment (Inzitari et al., 2008; Hao et al., 2013; Choi et al., 2019). One of the main functions of Hb is to input enough oxygen to the vascular nerve unit to meet basic nutritional requirements and output carbon dioxide, a local metabolite. Physiological or pathological changes in Hb are strongly correlated with erythrocyte function (Lin et al., 2018; in this study, the Spearman correlation coefficient was 0.859, *P* < 0.001). However, the effective components of erythrocytes in the central nervous system are not limited to Hb. The aforementioned cholinesterase activity, amyloid-β and α-synuclein are closely related to cognitive impairment (von Bernhardi et al., 2005; Lauriola et al., 2018; Graham et al., 2019).

Therefore, is there an association between erythrocyte count and VCI severity? To limit the scope of comparison, 302 patients were divided into mWMH and sWMH groups based on the severity of WMH. After single factor analyses, it was found that there were significant differences in Hb, hs-CRP, Hcy, β_2_-M, and PA levels between the two groups. The first three have been reported in the previous literature (Inzitari et al., 2008; Hilal et al., 2018; Wolters et al., 2019; Yang et al., 2020; Hirao et al., 2021; Wang X. et al., 2021), while the role of β_2_-M and PA in the formation of WMH remains unclear. Surprisingly, erythrocyte count, which was strongly correlated with Hb, was not significantly different between different grades of WMH.

Therefore, we further conducted multivariable analyses with patients with different severities of WMH and found that hs-CRP may be a common potential marker of different VCI levels in all patients with white matter injury. Inflammation is involved in vascular endothelial injury and nerve fiber demyelination, which is one of the main pathophysiological mechanisms of white matter degeneration (Hilal et al., 2018; Wang T. et al., 2020; Guo et al., 2021; Yang et al., 2021). As a nonspecific inflammatory marker, hs-CRP was significantly associated not only with VCI but also with the severity of white matter degeneration (Hilal et al., 2018; Boots et al., 2020), further confirming the structural basis of the inflammatory response of VCI. Importantly, the potential diagnostic value of erythrocyte, Hb, RBP, and PA levels for different cognitive levels was confirmed again in the sWMH group, but this was not observed in the mWMH group. This is a very interesting phenomenon, especially the erythrocyte count. Therefore, we took the sWMH group as the research object, and the ROC curve showed that erythrocyte counts were slightly more valuable in the diagnosis of sVCI than mVCI, suggesting that erythrocyte counts may have application value in the identification of different cognitive impairments in patients with sWMH. This has not been previously reported in the literature.

To further analyze the predictive value of multiple variables for different grades of VCI, an ordinal logistic regression model was created. Erythrocytes were found to be an independent protective factor that alleviates VCI. Even after adjusting for age, the independent effect of erythrocytes was still clear; however, after adjusting for sex, this effect disappeared, which suggested that predicting VCI cannot ignore the potential role of sex classification. To the best of our knowledge, this is the first report of this discovery. As mentioned above, vascular-derived cognitive impairment has a physical mechanism involving sudden neural circuit breakage and/or a biochemical mechanism mediated by chronic vascular endothelial injury. To adjust for the confounding effects caused by stroke lesions in the neural circuit, we classified and counted the lesions of patients with different cognitive levels as a whole and within subgroups. The results showed that the distribution of lesions was different, which is consistent with the maintenance of normal cognitive function that depends on the integrity of the neural network (Robertson, 2014; Escobar et al., 2019). The negative roles of parietal lobe lesions and atrial fibrillation in white matter degeneration or cognitive impairment have been confirmed (Robertson, 2014; Kato et al., 2016; Zhu et al., 2021b). Therefore, we included these two variables together with serum markers to construct the prediction model. Due to the strong correlation between Hb and erythrocytes, Hb was not included in this model.

How do erythrocytes have a cognitive protective effect in the neural network of sWMH patients? Although Hb is the main component of erythrocytes and its oxygen transport is essential for the normal function of vascular nerve units, no independent protective effect of Hb was found in this study. Erythrocytes are not just circulating organelles carrying nutrients. The complement receptors on the membrane surface can also mediate immune adhesion and phagocytosis, which play an important role in AD (Brubaker et al., 2017; Grzywa et al., 2021). Studies have found that increased erythrocyte-associated cholinesterase activity (von Bernhardi et al., 2005) and magnesium (Sitzia et al., 2020) had a protective effect on cognitive function, while Aβ42 (Lauriola et al., 2018), alpha-synuclein oligomer (Graham et al., 2019), hydroacetadedienoic acid and oxidatively modified peroxiredoxin (Yoshida et al., 2009) had the opposite effect. In addition, studies also showed that compared with the healthy control population, the increase of mean corpuscular volume (Gong et al., 2020), and the uncoupling disorder caused by the increase of capillary erythrocyte velocity in patients with VCI may damage the exchange of oxygen and nutrients (Zhang et al., 2019), thus aggravating the white matter damage. Therefore, in addition to the neuroprotective effect of Hb itself, we speculate that the non-hemoglobin components in the cytoplasm or the interaction of multiple components may be the basis for the protection of erythrocytes, and the immune regulation mediated by membrane receptors is worthy of further exploration.

This study has the following limitations: (1) although some of the elderly patients in our study had no behavioral symptoms of dementia, most patients were not screened for early manifestations of cognitive impairment of other types before the stroke. (2) The classification of white matter degeneration involved semiquantitative data, which are not as accurate as measuring WMH volume. (3) The sample size of CSVD patients was small. Although there were adjustments for stroke lesions, there was still the possibility of bias in the results. To carry out prospective pre-stroke comprehensive cognitive screening and design a multicenter longitudinal cohort study for high-risk populations in the community, accurately quantifying WMH volume will be more helpful for interpreting the conclusions of the study.

In conclusion, this study found that erythrocyte, Hb, hs-CRP, RBP, and PA levels are potential blood biomarkers of different cognitive levels in sWMH patients. Increased erythrocyte count is an independent protective factor for reducing the occurrence of VCI in patients with sWMH. Promoting hematopoietic function has great potential value for the prevention of cognitive decline in patients with CVD.

## Data Availability Statement

The raw data supporting the conclusions of this article will be made available by the authors, without undue reservation.

## Ethics Statement

The studies involving human participants were reviewed and approved by The ethics committee of Hunan Provincial People’s Hospital of Hunan Normal University. The patients/participants provided their written informed consent to participate in this study.

## Author Contributions

XT, DM, and QW: conceived and designed the study. XT, HZ, DM, SW, WT, CY, YZ, and QW: performed the study. XT, WZ, YL, ZC, CY, and QW: revised the article for intellectual content. XT and QW: wrote the article. All authors contributed to the article and approved the submitted version.

## Conflict of Interest

The authors declare that the research was conducted in the absence of any commercial or financial relationships that could be construed as a potential conflict of interest.

## Publisher’s Note

All claims expressed in this article are solely those of the authors and do not necessarily represent those of their affiliated organizations, or those of the publisher, the editors and the reviewers. Any product that may be evaluated in this article, or claim that may be made by its manufacturer, is not guaranteed or endorsed by the publisher.

## Abbreviations

AD: Alzheimer’s disease
ALP: alkaline phosphatase
AUC: area under the ROC curve
BMI: body mass index
BNT: Boston Naming Test
β_2_-M: β_2_ microglobulin
CHD: coronary heart disease
CDT: Clock Drawing Test
Cr: creatinine
CSVD: cerebral small vessel disease
CVD: cerebrovascular disease
DWMH: deep white matter hyperintensities
Hb: hemoglobin
Hcy: homocysteine
HDL-C: high-density lipoprotein cholesterol
hs-CRP: high-sensitivity C-reactive protein
HVLT-R: Hopkins Verbal Language Learning Lest-Revised
IMA: ischemia modified albumin
LDL-C: low-density lipoprotein cholesterol
MBI: modified Barthel index
MMSE: Mini Mental State Examination
mVCI: mild vascular cognitive impairment
mWMH: mild white matter hyperintensities
NC: normal cognition
PA: prealbumin
PWMH: periventricular white matter hyperintensities
RBP: retinol binding protein
ROC: receiver operating characteristic
sWMH: severe white matter hyperintensities
sVCI: severe vascular cognitive impairment
TG: triglycerides
TMT-A: Trail Making Test
UN: urea nitrogen
VCI: vascular cognitive impairment
VLDL-C: very low-density lipoprotein cholesterol
WMH: white matter hyperintensities.
