# Supplementary material for: Erythrocytes Are an Independent Protective Factor for Vascular Cognitive Impairment in Patients With Severe White Matter Hyperintensities
Source: Front Aging Neurosci. 2022 Feb 18;14:789602. doi: 10.3389/fnagi.2022.789602 (PMC8894857; doi:10.3389/fnagi.2022.789602)
Supplement: Supplementary file 1 [file Table_1.docx]

**Supplementary Table 1 Basic demographic data of patients with different cognitive levels**

| **Variables** | | **NC**  **(n=98)** | **mVCI (n=103)** | **sVCI (n=101)** | ***F/Χ^2^/H*** | ***P*** |
| --- | --- | --- | --- | --- | --- | --- |
| Age, years (Mean ±SD) | | 58.27 ± 12.36 | 66.21 ± 11.68 | 67.22 ± 11.18 | 17.302 | **0.000^***^** |
| BMI (Mean ±SD) | | 24.21 ± 2.82 | 23.59 ± 2.98 | 22.79 ± 3.40 | 5.334 | **0.005^**^** |
| Sex (male), n (%) | | 73 (74.49) | 70 (67.96) | 62 (61.39) | 3.918 | 0.141 |
| Education, n (%) | |  |  |  |  |  |
| 0 | | 0 (0.00) | 5 (4.85) | 6 (5.94) | 8.272 | **0.016^*^** |
| <6 | | 13 (17.81) | 24 (23.30) | 21 (20.79) |  |  |
| ≥7 | | 85 (86.73) | 74 (71.84) | 74 (73.27) |  |  |
| Stroke | |  |  |  |  |  |
|  | Ischemic stroke | 60 (61.22) | 64 (62.14) | 76 (75.25) | 5.541 | 0.063 |
|  | Hemorrhagic stroke | 11 (11.22) | 16 (15.53) | 20 (19.80) | 2.785 | 0.248 |
|  | Disease duration ^a^ | 1.60 (6.56) | 1.50 (4.27) | 2.00 (6.32) | 0.197 | 0.906 |
| CSVD, n (%) | | 29 (29.59) | 24 (23.30) | 9 (8.91) | 13.775 | **0.001^**^** |
| History of stroke, n (%) | | 3 (3.06) | 4 (3.88) | 5 (4.95) | 0.469 | 0.791 |
| Hypertension, n (%) | | 74 (75.51) | 81 (78.64) | 84 (83.17) | 1.790 | 0.409 |
| Diabetes mellitus, n (%) | | 37 (37,76) | 39 (37.86) | 41 (40.59) | 0.220 | 0.896 |
| CHD, n (%) | | 20 (20.41) | 27 (26.21) | 32 (31.68) | 3.274 | 0.195 |
| Atrial fibrillation, n (%) | | 3 (3.06) | 3 (2.91) | 13 (12.87) | 11.146 | **0.004^**^** |
| Smoking, n (%) | | 39 (39.80) | 33 (32.04) | 28 (27.72) | 3.355 | 0.187 |
| Alcohol intake, n (%) | | 23 (23.47) | 14 (13.59) | 18 (17.82) | 3.305 | 0.192 |

^*^*P*˂0.05, ^**^ *P*˂0.01, ^***^ *P*˂0.001. ^a^ shown as median (IQR).

*Abbreviations*: BMI, body mass index; CHD, Coronary heart disease; CSVD, cerebral small vessel disease; mVCI, mild vascular cognitive impairment; NC, normal cognition; SD, standard deviation; sVCI, severe vascular cognitive impairment.

**Supplementary Table 2 Comparison of blood markers and behavioral scores among the three groups**

| **Variable** | **NC (n=98)** | **mVCI (n=103)** | **sVCI (n=101)** | ***F / H*** | ***P*** | **Tukey / Bonferron adjusted** | | |
| --- | --- | --- | --- | --- | --- | --- | --- | --- |
|  |  |  |  |  |  | **NC *vs.* mVCI** | **NC *vs.* sVCI** | **mVCI *vs.* sVCI** |
| Erythrocyte (× 10^12^/L) ^a^ | 4.45 ± 0.54 | 4.25 ± 0.52 | 4.15 ± 0.56 | 7.843 | **0.000^***^** | **0.027^*^** | **0.000^***^** | 0.374 |
| Hb (g/L) ^b^ | 134.50 (22.50) | 128.00(25.00) | 128.00(23.5) | 11.627 | **0.003^**^** | 0.072 | **0.002^**^** | 0.793 |
| Platelets (× 10^9^/L) ^b^ | 209.00(75.25) | 217.00(76.00) | 226.00(92) | 4,794 | 0.091 | / | / | / |
| Lymphocytes(×10^9^/L) ^b^ | 1.81(0.75) | 1.65(0.80) | 1.48(0.66) | 13.130 | **0.001^**^** | 0.427 | **0.001^**^** | 0.091 |
| hs-CRP (mg/L)^b^ | 1.24(2.23) | 2.34(4.34) | 4.41(8.99) | 28.606 | **0.000^***^** | **0.033^*^** | **0.000^***^** | **0.013^*^** |
| Hcy (μmol/L) ^b^ | 13.92(6.51) | 14.66(5.68) | 15.20(6.35) | 1.181 | 0.554 | / | / | / |
| RBP (mg/L) ^b^ | 39.05(11.15) | 38.40(14.10) | 33.80(14.40) | 7.632 | **0.022^*^** | 1.000 | **0.044^*^** | 0.058 |
| Cr (μmol/L) ^b^ | 69.00(23.25) | 67.00(30.00) | 65.00(26.00) | 2.592 | 0.274 | / | / | / |
| UN (mmol/L) ^b^ | 5.18(1.63) | 5.20(1.96) | 5.27(2.39) | 0.105 | 0.949 | / | / | / |
| β_2_-M (mg/L) ^b^ | 2.07(0.81) | 2.39(0.87) | 2.30(0.87) | 12.114 | **0.002^**^** | **0.005^**^** | **0.011^*^** | 1.000 |
| ALP (U/L) ^b^ | 67.00(23.00) | 71.00(21.00) | 71.00(24.5) | 3.870 | 0.144 | / | / | / |
| TG (mmol/L) ^b^ | 1.42(0.97) | 1.33(0.92) | 1.33(0.90) | 0.256 | 0.880 | / | / | / |
| HDL-C (mmol/L) ^b^ | 1.04(0.32) | 1.04(0.42) | 0.97(0.37) | 3.431 | 0.180 | / | / | / |
| LDL-C (mmol/L) ^b^ | 2.43(1.48) | 2.28(1.14) | 2.22(1.12) | 1.255 | 0.534 | / | / | / |
| VLDL-C (mmol/L) ^b^ | 0.39(0.27) | 0.36(0.25) | 0.36(0.29) | 1.117 | 0.572 | / | / | / |
| IMA (U/mL) ^b^ | 76.60(5.90) | 77.70(4.20) | 78.50(5.20) | 8.170 | **0.017^*^** | 0.378 | **0.013^*^** | 0.530 |
| PA (mg/L) ^a^ | 270.12 ± 61.41 | 260.93 ± 61.62 | 237.52 ± 67.85 | 6.967 | **0.001^**^** | 0.563 | **0.001^**^** | **0.025^*^** |
| Total scores of WMH ^b^ | 2.00(2.00) | 3.00(3.00) | 3.00(2.00) | 29.705 | **0.000^***^** | **0.002^**^** | **0.000^***^** | 0.126 |
| Scores of PWMH ^b^ | 1.00(0.25) | 1.00(1.00) | 2.00(1.00) | 35.679 | **0.000^***^** | **0.001^**^** | **0.000^***^** | 0.086 |
| Scores of DWMH ^b^ | 1.00(1.00) | 1.00(1.00) | 1.00(1.00) | 17.278 | **0.000^***^** | **0.031^*^** | **0.000^***^** | 0.337 |
| MMSE ^b^ | 28.50(2.00) | 27.00(3.00) | 18.00(10.50) | 196.576 | **0.000^***^** | **0.000^***^** | **0.000^***^** | **0.000^***^** |
| CDT ^b^ | 4.00(0.00) | 4.00(1.00) | 2.00(2.00) | 203.902 | **0.000^***^** | **0.000^***^** | **0.000^***^** | **0.000^***^** |
| BNT ^b^ | 24.00(2.00) | 16.00(3.00) | 15.00(12.00) | 179.869 | **0.000^***^** | **0.000^***^** | **0.000^***^** | **0.000^***^** |
| HVLT-R ^b^ | 21.00(2.00) | 16.00(3.00) | 10.00(10.00) | 228.597 | **0.000^***^** | **0.000^***^** | **0.000^***^** | **0.000^***^** |
| MBI ^b^ | 97.50(21.25) | 80.00(45.00) | 55.00(50.00) | 69.655 | **0.000^***^** | **0.000^***^** | **0.000^***^** | **0.000^***^** |

^*^*P*˂0.05, ^**^ *P*˂0.01, ^***^ *P*˂0.001. ^a^ shown as mean ± standard deviation. ^b^ shown as median (IQR).

*Abbreviations*: ALP, alkaline phosphatase; BNT, Boston Naming Test; β_2_-M, β_2_ microglobulin; CDT, Clock Drawing Test; Cr, creatinine; Hb, hemoglobin; Hcy, homocysteine; HDL-C, high-density lipoprotein cholesterol; hs-CRP, high-sensitivity C-reactive protein; HVLT-R, Hopkins Verbal Language Learning Lest-Revised; IMA, ischemia modified albumin; LDL-C, low-density lipoprotein cholesterol; MBI, modified Barthel index; MMSE, Mini Mental State Examination; mVCI, mild vascular cognitive impairment; NC, normal cognition; PA, prealbumin; RBP, retinol binding protein; sVCI, severe vascular cognitive impairment; TG, triglycerides; TMT-A, Trail Making Test; UN, urea nitrogen; VLDL-C, very low-density lipoprotein cholesterol; WMH, white matter hyperintensities.

**Supplementary Table 3 Comparison of lesion locations among the different groups**

| **Variables ^a^** | **Groups** | **NC** | **mVCI** | **sVCI** | ***Χ^2^ / H*** | ***P*** |
| --- | --- | --- | --- | --- | --- | --- |
| Brain atrophy | Total | 62(63.27) | 84(81.55) | 91(90.10) | 22.079 | **0.000^***^** |
|  | mWMH | 39(54.17) | 37(72.55) | 30(81.08) | 9.247 | **0.010^*^** |
|  | sWMH | 23(88.46) | 47(90.38) | 61(95.31) | 1.663 | 0.435 |
| Frontal lobe | Total | 15(15.31) | 21(20.39) | 33(32.67) | 9.047 | **0.011^*^** |
|  | mWMH | 11(15.28) | 7(13.73) | 18(48.65) | 18.914 | **0.000^***^** |
|  | sWMH | 4(15.38) | 14(26.92) | 15(23.44) | 1.296 | 0.523 |
| Parietal lobe | Total | 7(7.14) | 15(14.56) | 33(32.67) | 23.162 | **0.000^***^** |
|  | mWMH | 7(9.72) | 4(7.84) | 17(45.95) | 27.048 | **0.000^***^** |
|  | sWMH | 0(0.00) | 11(21.15) | 16(25.00) | 12.504 | **0.002^**^** |
| Temporal lobe | Total | 10(10.20) | 16(15.53) | 29(28.71) | 12.192 | **0.002^**^** |
|  | mWMH | 6(8.33) | 8(15.69) | 15(40.54) | 17.384 | **0.000^***^** |
|  | sWMH | 4(15.38) | 8(15.38) | 14(21.88) | 0.985 | 0.611 |
| Occipital lobe | Total | 7(7.14) | 12(11.65) | 22(21.78) | 9.579 | **0.008^**^** |
|  | mWMH | 5(6.94) | 6(11.76) | 12(32.43) | 13.314 | **0.001^**^** |
|  | sWMH | 2(7.69) | 6(11.54) | 10(15.63) | 1.202 | 0.548 |
| Insular lobe | Total | 4(4.08) | 6(5.82) | 12(11.88) | 4.973 | **0.044^*^** |
|  | mWMH | 2(2.78) | 4(7.84) | 7(18.92) | 7.966 | **0.019^*^** |
|  | sWMH | 2(7.69) | 2(3.85) | 5(7.81) | 0.922 | 0.631 |
| Thalamus | Total | 9(9.18) | 8(7.77) | 17(16.83) | 4.819 | 0.090 |
|  | mWMH | 4(5.56) | 1(1.96) | 6(16.21) | 6.586 | **0.037^*^** |
|  | sWMH | 5(19.23) | 7(13.46) | 11(17.19) | 0.515 | 0.773 |
| Cerebellum | Total | 7(7.14) | 6(5.83) | 10(9.90) | 1.250 | 0.535 |
|  | mWMH | 6(8.33) | 3(5.88) | 2(5.41) | 0.441 | 0.802 |
|  | sWMH | 1(3.85) | 3(5.77) | 8(12.50) | 2.615 | 0.270 |
| Hippocampus | Total | 0(0.00) | 3(2.91) | 3(2.97) | 4.767 | 0.092 |
|  | mWMH | 0(0.00) | 3(5.88) | 3(8.11) | 7.530 | **0.023^*^** |
|  | sWMH | 0(0.00) | 0(0.00) | 0(0.00) | / | / |
| Brain stem | Total | 19(19.39) | 19(18.45) | 18(17.82) | 0.082 | 0.960 |
|  | mWMH | 12(16.67) | 7(13.73) | 4(10.81) | 0.707 | 0.702 |
|  | sWMH | 7(26.92) | 12(46.15) | 14(21.88) | 0.265 | 0.876 |
| Basal ganglia | Total | 25(28.57) | 32(31.07) | 45(44.55) | 8.577 | **0.014^*^** |
|  | mWMH | 18(25.00) | 15(29.41) | 13(35.14) | 1.242 | 0.538 |
|  | sWMH | 7(26.92) | 17(65.38) | 32(50.00) | 5.685 | 0.058 |
| Lateral ventricle | Total | 4(4.08) | 8(7.77) | 10(9.90) | 2.548 | 0.280 |
|  | mWMH | 3(4.17) | 2(3.92) | 1(2.70) | 0.162 | 0.922 |
|  | sWMH | 1(3.85) | 6(11.54) | 9(14.06) | 2.338 | 0.311 |
| Corona radiata | Total | 11(11.22) | 14(13.59) | 10(9.90) | 0.697 | 0.706 |
|  | mWMH | 9(12.50) | 7(13.73) | 5(13.51) | 0.046 | 0.977 |
|  | sWMH | 2(7.69) | 14(26.92) | 5(7.81) | 9.229 | **0.010^*^** |
| Centrum ovale | Total | 4(4.08) | 3(2.91) | 7(6.93) | 1.915 | 0.384 |
|  | mWMH | 2(2.78) | 0(0.00) | 2(5.41) | 3.571 | 0.168 |
|  | sWMH | 2(7.69) | 3(5.77) | 5(7.81) | 0.209 | 0.901 |
| Corpus callosum | Total | 1(1.02) | 5(4.85) | 8(7.92) | 6.253 | **0.044^*^** |
|  | mWMH | 1(1.39) | 2(3.92) | 3(8.11) | 2.935 | 0.231 |
|  | sWMH | 0(0.00) | 3(5.77) | 5(7.81) | 3.530 | 0.171 |

^*^*P*˂0.05, ^**^ *P*˂0.01, ^***^ *P*˂0.001. ^a^ shown as n (%).

*Abbreviations*: mVCI, mild vascular cognitive impairment; NC, normal cognition; sVCI, severe vascular cognitive impairment.

**Supplementary Table 4 Comparison of various biomarkers from those with different cognitive levels in the mWMH group**

| **Variables** | **NC (n=72)** | **mVCI (n=51)** | **sVCI (n=37)** | ***F / H*** | ***P*** | **Tukey / Bonferron adjusted** | | |
| --- | --- | --- | --- | --- | --- | --- | --- | --- |
|  |  |  |  |  |  | **NC *vs.* mVCI** | **NC *vs.* sVCI** | **mVCI *vs.* sVCI** |
| Erythrocyte (× 10^12^/L) ^a^ | 4.40(0.81) | 4.19(0.91) | 4.19(0.53) | 2.993 | 0.224 | / | / | / |
| Hb (g/L) ^a^ | 134.00(23.50) | 130.00(27.00) | 129.00(14.5) | 2.405 | 0.300 | / | / | / |
| Platelets (× 10^9^/L) ^a^ | 209.00(88.50) | 203.00(73.00) | 235.00(100.00) | 3.469 | 0.177 | / | / | / |
| Lymphocytes(×10^9^/L) ^a^ | 1.74(0.83) | 1.76(0.98) | 1.41(0.63) | 9.942 | **0.007^**^** | 1.000 | **0.010^*^** | **0.020^*^** |
| hs-CRP (mg/L) ^a^ | 1.24(2.17) | 2.39(5.43) | 3.23(7.53) | 12.411 | **0.002^**^** | 0.059 | **0.003^**^** | 0.764 |
| Hcy (μmol/L) ^a^ | 13.28(6.24) | 14.90(7.24) | 13.94(5.52) | 0.710 | 0.701 | / | / | / |
| RBP (mg/L) ^a^ | 38.45(13.67) | 38.70(13.5) | 35.8(14.7) | 0.944 | 0.624 | / | / | / |
| Cr (μmol/L) ^a^ | 65.50(20.75) | 67.00(30.00) | 60.00(23.5) | 6.817 | **0.033^*^** | 1.000 | 0.146 | **0.031^*^** |
| UN (mmol/L) ^a^ | 5.33(1.77) | 5.54(2.72) | 5.10(2.41) | 1.794 | 0.408 | / | / | / |
| β_2_-M (mg/L) ^a^ | 2.03(0.70) | 2.29(0.70) | 2.10(0.84) | 3.811 | 0.419 | / | / | / |
| ALP (U/L) ^a^ | 67.50(22.50) | 71.00(22.00) | 69.00(37.00) | 1.260 | 0.533 | / | / | / |
| TG (mmol/L) ^a^ | 1.42(0.96) | 1.37(0.94) | 1.39(0.98) | 0.661 | 0.718 | / | / | / |
| HDL-C (mmol/L) ^a^ | 1.03(0.32) | 1.15(0.36) | 0.95(0.36) | 8.095 | **0.017^*^** | 0.708 | 0.153 | **0.014^*^** |
| LDL-C (mmol/L) ^a^ | 2.46(1.51) | 2.54(1.31) | 2.11(1.06) | 2.119 | 0.347 | / | / | / |
| VLDL-C (mmol/L) ^a^ | 0.39(0.27) | 0.40(0.27) | 0.39(0.27) | 0.474 | 0.789 | / | / | / |
| IMA (U/mL) ^b^ | 76.43±3.70 | 77.73±4.17 | 77.78±4.75 | 2.049 | 0.132 | / | / | / |
| PA (mg/L) ^a^ | 265.00(82.85) | 256.20(80.30) | 261.80(67.30) | 0.738 | 0.691 | / | / | / |

^*^*P*˂0.05, ^**^ *P*˂0.01, ^***^ *P*˂0.001. ^a^ shown as median (IQR). ^b^ shown as Mean ± standard deviation.

*Abbreviations*: ALP, alkaline phosphatase; β_2_-M, β_2_ microglobulin; Cr, creatinine; Hb, hemoglobin; Hcy, homocysteine; HDL-C, high-density lipoprotein cholesterol; hs-CRP, high-sensitivity C-reactive protein; IMA, ischemia modified albumin; LDL-C, low-density lipoprotein cholesterol; mVCI, mild vascular cognitive impairment; mWMH, mild white matter hyperintensities; NC, normal cognition; PA, prealbumin; RBP, retinol binding protein; sVCI, severe vascular cognitive impairment; TG, triglycerides; UN, urea nitrogen; VLDL-C, very low-density lipoprotein cholesterol.
